# Case Report of an Atypical Abdominal Pain Found to be a Rare Corynebacterium Emphysematous Pyelitis

**DOI:** 10.7759/cureus.23579

**Published:** 2022-03-28

**Authors:** Manpreet Singh, Obed Barrera Adame, Mehrdad Alaie

**Affiliations:** 1 Emergency Department, St. Barnabas Hospital (SBH) Health System, Bronx, USA

**Keywords:** case report, nephrectomy, corynebacterium, pyelonephritis, pyelitis

## Abstract

Recognizing life-threatening infections is crucial for an emergency physician. In this case report, we describe an atypical presentation of a severe, infiltrating kidney infection, which, if not recognized early, could have led to a detrimental outcome. Emphysematous pyelitis, which is class I of emphysematous pyelonephritis, is a rare entity, and patients may present with a urinary tract infection or pyelonephritis. To add to this, underrecognition of gram-positive organisms, in this case *Corynebacterium*, can delay treatment and worsen outcomes, as described in this case. Through this case, we wish to create awareness of this disease and to reinforce emergency physicians to keep this entity on their differential diagnosis when evaluating an immunocompromised patient.

## Introduction

Emphysematous pyelitis is a relatively uncommon, acute necrotizing infection of the renal system. Huang and Tseng classify emphysematous pyelitis as class I of IV classes of emphysematous pyelonephritis, with class I showing gas in the collecting system and class IV compromising of bilateral necrotizing infection of renal parenchyma [1]. Research shows that 90% of emphysematous pyelonephritis cases are seen in patients with uncontrolled diabetes and in those with urinary tract obstruction, with *Escherichia Coli* identified as being the most commonly isolated organism [1,2].

Emphysematous pyelonephritis carries a high mortality, and therefore early recognition is crucial. It usually presents with vague complaints, and if left untreated, it leads to a fulminant course with mortality ranging from 20% for class I to 70% for class IV [3]. Here, we describe a rather unusual case of a middle-aged female who presented to the emergency department with a complaint of chronic abdominal pain for months but with acutely worsening symptoms.

## Case presentation

The patient is a 45-year-old female who presented to the emergency department with the chief complaint of abdominal pain. She reported that her abdominal pain initially began about three months ago and has been intermittent since then. The current episode started a day ago but worsened about an hour prior to her presentation to the emergency department. The abdominal pain was described as sharp and localized to her left upper quadrant with radiation to her back and was associated with nausea and dysuria. At presentation, she denied any associated vomiting, fever, chills, chest pain, shortness of breath, hematuria, diarrhea, or constipation. The patient’s past medical history was only significant for type 2 diabetes.

The patient's physical examination revealed a temperature of 37.7 degrees Celsius, heart rate of 97 beats per minute, blood pressure of 98/61mm Hg, and respiratory rate of 18 breaths per minute with oxygen saturation of 100% on room air. Her skin was warm, and her lungs were clear to auscultation bilaterally. Her cardiac examination showed a regular rate and rhythm without any murmurs. The abdomen was soft, non-distended but diffusely tender to palpation with most tenderness localized to the left upper quadrant with positive left costovertebral angle tenderness, and bowel sounds were present with no rebound or guarding.

Laboratory analysis included a urinalysis, which showed glucose of 150 mg/dL (reference range: 0-15 mg/dL), positive nitrite and leukocyte esterase with white blood cell count of 95/HPC (0-5/HPF). Other significant results included white blood cell count of 15.1 x 10^3^/uL (4.2-9.1 x 10^3^/uL), neutrophils 81.7% (34-67.9%), potassium of 2.0 mEq/L (3.5-5.3 mEq/L), urea nitrogen of 5 mg/dL (8-23 mg/dL), creatinine of 1.5 mg/dL (0.6-1.2 mg/dL), magnesium of 0.9 mEq/L (1.3-2.1 mEq/L), and lactate of 9.1 mmol/L (0.0-2.0 mmol/L). The patient's blood glucose was 308 mg/dL (70-99 mg/dL). Coagulation studies were within normal limits.

**Figure 1:**
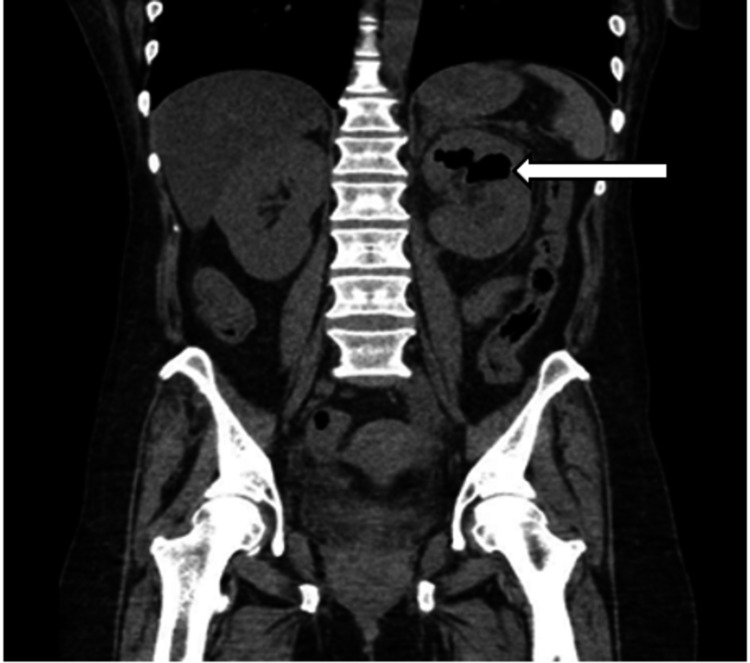
CT the abdomen and pelvis without contrast The white arrow demonstrates left hydronephrosis with gas within the left renal collecting system with possible extension into renal parenchyma.

**Figure 2:**
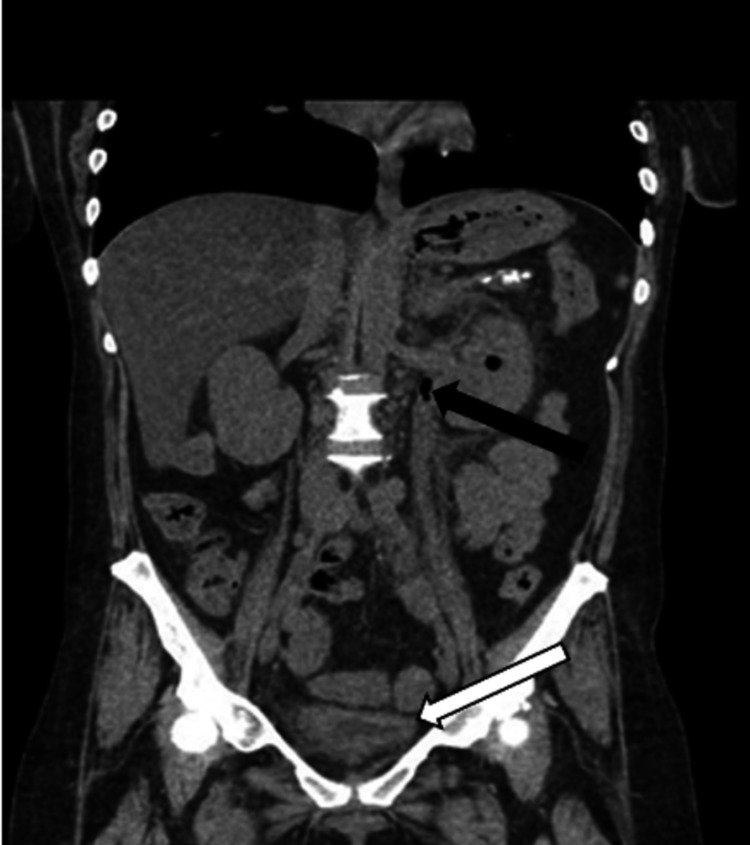
CT of the abdomen and pelvis without contrast Abdominal CT demonstrating gas in the renal collecting system, with the black arrow demonstrating gas in the ureter with hydroureter and the white arrow showing thickened bladder wall with perivesicular edema.

On re-evaluation, the patient complained of worsening abdominal pain and was noted to be tachycardic, which prompted the team to obtain a computed tomography (CT) of the abdomen and pelvis without contrast. The CT revealed left hydronephrosis with gas within the left renal collecting system concerning for emphysematous pyelitis (Figure 1). It also showed mild left hydroureter extending to the level of the urinary bladder, a thickened bladder wall, and perivascular edema concerning for ascending bladder infection (Figure 2). At this point, an emergent urology consultation was requested, and the patient was started on 1 gram of ceftriaxone and 4.5 grams of piperacillin/tazobactam, along with intravenous (IV) fluid resuscitation with normal saline and electrolyte replacement therapy for her hypomagnesemia and hypokalemia. While awaiting urology consultation, the patient’s clinical presentation was noted to be deteriorating with increased tenderness in the left upper quadrant, persistent tachycardia (105-115s), and a fever of 38.1°C. Despite fluid resuscitation, the patient developed hypotension with blood pressure less than 90s/60s mm Hg and was placed on norepinephrine infusion. Now with the development of urosepsis, the rapid progression to emphysematous pyelonephritis was suspected. After the patient was seen and evaluated by urology, she was taken emergently to the operating room for ureter stenting. Unfortunately, ureter stenting failed, and the patient was taken emergently by interventional radiology for a left nephrostomy tube placement. She was admitted to the intensive care unit for septic shock requiring pressor support and significant electrolyte replacement therapy.

Urine culture showed positive *E. coli*, and nephrostomy tube aspirate culture grew *Corynebacterium*, *E. coli*, and *Streptococcus anginosus*. After a complicated but successful hospital course, the patient was discharged. A CT scan of the abdomen was repeated 36 days after the initial finding of emphysematous pyelitis, which showed a complete resolution of the initial CT findings. She had her nephrostomy tube removed after 37 days from placement without any further complication.

## Discussion

The renal system is comprised of the kidneys, ureters, bladder, and urethra. The presence of air in a naive renal system alludes to a severe infection. Emphysematous urinary tract infections are a rare event and carry a high mortality, and the pathogenesis is still not fully understood [4]. Emphysematous pyelitis is a gas-producing acute bacterial infection isolated to the renal collecting system [5]. Huang and Tseng classify emphysematous pyelitis as I of IV classes of emphysematous pyelonephritis, with class I having gas the collecting systems and class IV having bilateral necrotizing emphysematous pyelonephritis with parenchymal destruction. Another classification by Wan et al. categorize emphysematous pyelitis as type II emphysematous pyelonephritis, where gas or fluid can be found in the renal or perirenal collective system [1,6]. The main risk factors for emphysematous pyelitis include woman gender and the presence of diabetes mellitus, which is found in approximately 90% of cases [7]. Urinary bacterial cultures commonly include *E. coli* (69%), *Klebsiella pneumoniae* (29%), and rarely *Aerobacter aerogenes* and *Proteus mirabilis* [2].

What makes this case unique is that even though urinary culture grew just E. coli, the patient’s direct renal percutaneous aspirate performed by interventional radiology grew *Corynebacterium*, *E. coli*, and *S. anginosus*. Although the subtype of Corynebacterium was unable to be obtained, the presence of this organism can postulate an explanation for the rapid clinical deterioration of our patient. *Corynebacterium urealyticum* is a gram-positive, slow-growing, lipophilic, multi-drug resistant, urease-positive microorganism with diphtheroid morphology [8]. Although previously Corynebacterium was thought to be a contaminant of skin flora due to its wide distribution in skin and mucous membrane, it is now identified as an opportunistic pathogen mainly involved in acute cystitis, pyelonephritis, alkaline encrusted cystitis, encrusted pyelitis, and, in rare occasions, bacteremia. Rizvi et al. reported the incidence of *Corynebacterium urealyticum* to be 1-2% in the general population [9].

Diagnosis is made via CT, which is the imaging of choice and is considered the most sensitive and specific modality for the evaluation of emphysematous pyelonephritis [10]. Classification and staging of emphysematous pyelonephritis are based on radiological findings of the extension/presence of air in the collecting system and calyx [11]. CT allows for the evaluation of the renal parenchyma as well as to differentiate between gas, fluids, and stones. It can also precisely localize where the abnormality is present, allowing for a detailed evaluation of the renal parenchyma and/or pararenal spaces. This allows for an accurate diagnosis and staging of the different classes of emphysematous pyelonephritis, which is crucial since the initial treatment has a large variation [12]

Emphysematous pyelonephritis presents with vague complaints of flank pain, fevers, and chills. The initial treatment is complicated as it always involves clinical features, radiological classifications, comorbidities, and prognostic factors [3]. Current recommendations for uncomplicated emphysematous pyelitis involve management with antibiotics, fluid resuscitation, electrolyte replacement, glucose control, and alleviation of obstruction as needed. Antibiotics management should target gram-negative bacteria; however, as seen in this case, gram-positive bacteria cannot be excluded. If the disease has progressed to complicated pyelonephritis and ureteric obstruction is present, percutaneous nephrostomy or stent is recommended [2]. For cases where the disease has progressed to class IV emphysematous pyelonephritis with diffuse gas present within the renal parenchyma with associated destruction, nephrectomy is advised [2,12].

Despite all the advances in medicine, emphysematous pyelonephritis has a fulminant course, rapid deterioration, and fatal outcomes if left untreated [2,3]. As noted in this case, ultimately treatment should be tailored to the patient’s current clinical presentation.

## Conclusions

Emphysematous pyelitis is an uncommon infection of the upper urinary tract with a high mortality rate and an unpredictable course. Diagnosis is made via CT and treatment varies depending on the stage. This case illustrated the sudden deterioration in an emphysematous pyelitis patient, who are typically treated with antibiotics alone, but here required emergent surgical intervention attributable to rapid disease progression to septic shock requiring pressors and aggressive electrolyte replacement therapy. In immunocompromised patients, emphysematous pyelitis should be in the differential diagnosis. The patient shown in this case had a rare and indolent clinical presentation. Paying attention to the patient’s immunocompromised state and unstable vital signs led to further workup, correct diagnosis, and aggressive treatment with an appropriate disposition.
